# GLP-1 is associated with perfectionism in Swedish women with anorexia nervosa, independent of BMI

**DOI:** 10.1007/s40519-026-01831-x

**Published:** 2026-03-03

**Authors:** Sofia Elm, Suzanne Petersson, Martin Carlsson, Lars Brudin, Ina Marteinsdottir, Pär Wanby

**Affiliations:** 1 Department of Pediatrics, County of Linköping, Linköping, Sweden; 2 Department of Medicine and Optometry, Linnaeus University, Kalmar, Sweden; 3 Department of Rehabilitation, Region Kalmar County, Kalmar, Sweden; 4 Department of Medical and Health Sciences, University of Linköping, Linköping, Sweden; 5 Department of Clinical Physiology, Region Kalmar County, Kalmar, Sweden; 6 Department of Psychiatry, University of Linköping, Region Kalmar County, Sweden; 7 Department of Internal Medicine, Section of Endocrinology, Kalmar County Hospital, 39244 Region Kalmar CountyKalmar, Sweden

**Keywords:** Anorexia nervosa, Incretins, GLP-1, Perfectionism

## Abstract

**Purpose:**

Based on the presence of early satiety in anorexia nervosa (AN), which may delay recovery, and given the dramatic impact of GLP-1 and GIP analog treatment on satiety and weight reduction in obesity by diminishing the sensation of hunger, we initiated this cross-sectional pilot study to explore fasting incretin levels in AN and to identify possible correlations between these hormones and psychiatric symptoms.

**Methods:**

17 female subjects aged 18–35 were enrolled; 10 previously diagnosed with AN (5 weight restored, 5 non-weight restored) and 7 healthy controls (HC). Fasting blood samples were analyzed for incretin levels using ELISA. Psychiatric symptoms were evaluated using self-assessment scales for eating disorders (EDI-3, EDE-Q), anxiety (STAI-S + T), depression (MADRS-S), and obsessive–compulsive disorder (OCI-R).

**Results:**

Subjects with AN scored overall higher on psychiatric scales, indicating poorer psychological well-being than HC. In non-weight restored AN (non-WRAN) subjects both serum GLP-1 levels (29 (15–85) vs 16 (15–21) pg/mL; *p* = 0.048) as well as serum GIP levels (37 (14–163) vs 5 (2–58) pmol/L; *p* = 0.048) were elevated compared to HC. No differences were found in glucagon or PYY levels between groups. A strong correlation between serum GLP-1 levels and EDI-perfectionism independent of BMI (r = 0.768, *p* = 0.001) was found in the entire group.

**Conclusions:**

Independent of BMI, GLP-1 levels were associated with perfectionism, a typical characteristic of AN. Fasting GLP-1 levels were elevated in subjects with non-WRAN. Elevated GLP-1 levels may perpetuate AN symptoms, emphasizing the need for further research into their role in the metabolic and psychiatric dimensions of AN.

*Level of evidence*: Level III: Evidence obtained from well-designed case–control analytic studies.

**Supplementary Information:**

The online version contains supplementary material available at 10.1007/s40519-026-01831-x.

## Introduction

Anorexia Nervosa (AN), is a complex metabolic-psychiatric disorder [1, 2] characterized by self-imposed starvation, abnormally low body weight, intense fear of gaining weight, and a distorted perception of body image. Individuals with AN often exhibit an excessive preoccupation with food, engage in ritualistic eating behaviors, and may display elevated levels of physical activity [3]. Other common psychological features include depression, anxiety, perfectionism, and emotional rigidity [4]. Perfectionism has been found to play a crucial role in the development and maintenance of anorexia nervosa [5, 6] and women exhibiting higher levels of perfectionism have been shown to display the most severe eating disorder symptoms [2].

The etiology of AN is multifaceted, involving a combination of genetic, psychological and environmental factors [7] to date not fully clarified. Interactions between genetic vulnerability and anxiety, perfectionism, and cognitive rigidity are postulated to play a role [8]. Recent genome-wide association studies suggest that metabolic factors are also implicated in the development of AN [9].

Metabolic factors involved in AN are characterized by a range of endocrine abnormalities [10], considered mostly to be adaptive responses that facilitate survival in a low-energy state induced by chronic starvation. Hormonal changes commonly observed in AN include hypothalamic amenorrhea, relative hypercortisolism, non-thyroidal illness syndrome, hypoleptinemia, reduced levels of insulin and amylin, and elevated levels of appetite regulating hormones such as peptide YY, ghrelin, and adiponectin. GLP-1 and GIP are released during meals from gastrointestinal L, respectively, K cell and, in addition to glucose dependently inducing insulin secretion potently through central mechanisms, reduce food intake by suppressing appetite. Incretins are dysregulated in obesity and type II diabetes, and treatment of these conditions with incretin agonists, with improved pharmacokinetic properties, are currently in clinical use. The most effective drugs to date consist of a combined GIP/GLP1 agonist (such as tirzepatide), which stimulates receptors for both of these incretins, resulting in dramatic weight loss [11]. Preliminary evidence also suggests that incretin-based therapies may reduce addictive behaviors [12].

Changes in incretins have been reported in AN [13], but studies on glucagon-like peptide-1 (GLP-1) and gastric inhibitory polypeptide (GIP) are limited and have yielded contradictory results [14, 15]. Apart from the recent study by Kolb and colleagues [16] which demonstrated a positive association between GIP, disordered eating, and depressive symptoms, the relationship between incretin hormones and psychiatric symptoms in anorexia nervosa (AN) has not previously been investigated.

Hormonal imbalances in AN [10] may influence neurocognitive functions, anxiety, depression, and other eating disorder (ED) psychopathologies [13]. Low levels of gonadal hormones, oxytocin, and leptin, along with high levels of cortisol, have been associated with heightened symptoms of depression and anxiety in AN [10]. Additionally, elevated levels of ghrelin and neuropeptide Y (NPY) have been linked to obsessiveness with food [17], while low leptin levels and high peptide YY (PYY) and ghrelin levels have been associated with drive for thinness [18].

Based on an early satiety in AN [19, 20], which may delay recovery and, given the dramatic impact on satiety and weight reduction in obesity using treatment with GLP-1 and GIP analogs [21, 22], we initiated this cross-sectional pilot study aiming to investigate basal incretin levels in AN and healthy controls and to identify possible correlations for these hormones with psychiatric symptoms in AN. PYY and cortisol have been correlated to psychopathologies in AN [13] and were therefore also analyzed.

## Materials and methods

### Study participants

Participants diagnosed with anorexia nervosa (AN) were identified through a computerized search conducted in January 2022 within the digital medical record system, Cambio Cosmic. This search identified all patients (n = 38), aged 16–35 years, who were diagnosed with AN (ICD code F50.0) between January 2016 and January 2019 or had been diagnosed earlier, but with the diagnosis still present. The study design aimed at including adult patients, but also patients aged > / = 16, considered biologically comparable to adults [10, 23]. All diagnoses had been made according to DSM-IV criteria [24] at either of two outpatient clinics in southeastern Sweden specializing in eating disorder treatment. Thirty of the 38 patients were still living in the region and were by letter invited to participate in the study. Of these, 17 responded to the invitation, but seven were not included due to the presence of one or more of the following conditions: severe anxiety, depressive conditions, psychotic disorders, active alcohol abuse, or a purging subtype of AN. Since purging may influence levels of appetite-regulating hormones we aimed at not including patients with purging behavior [25]. Participants who provided written informed consent, completed psychiatric self-assessment scales, and fasting blood samples for analysis of appetite-regulating hormones were included in the study. In total, ten women with a previous or current diagnosis of AN were included. Additionally, seven healthy blood donors of similar ages from the same geographic region, who completed the psychiatric self-assessment scales and provided fasting blood samples, were recruited as controls. Medical histories for both AN-diagnosed subjects and blood donors were collected through a nurse-conducted interview. All study participants were permitted to use estrogen therapy.

### Anthropometric measurements

Weight and height were measured with the subjects barefoot in light clothing, and Body Mass Index (BMI) was calculated as kilograms per square meter. The 10 subjects with a current or previous diagnosis of AN were further divided into two groups, weight restored AN (WRAN) if their BMI was > 18.5 kg/m^2^ and non-weight restored AN (non-WRAN), if their BMI < 18.5 kg/m^2^.

### Assays

Venous blood samples were drawn for analysis after an overnight fast. Serum samples of the appetite regulating hormones, glucagon-like peptide-1 (GLP-1), glucose-dependent insulinotropic polypeptide (GIP), glucagon (incretins), and peptide Y (PYY) were analyzed using enzyme-linked immunosorbent assay (ELISA). GLP-1, and glucagon were analyzed in triplicates according to manufacturer’s instructions (GLP-1 ELISA AL-172 and Glucagon ELISA AL-157-I; AnshLabs, Webster, Texas, USA). PYY was analyzed in triplicate according to manufacturer’s instructions (Yanaihara Institute Inc, Shizuoka, Japan).

All samples were analyzed in duplicate. The coefficients of variation were for all analyses > 15%. Values for GLP-1 < LOD (Limit of detection) 15 (pg/mL) were adjusted to 15, as values for GIP for > LOD were adjusted to 1.88 (pmol/L) and a Glucagon value > LOD was adjusted to 290 (pg/mL).

Plasma cortisol and plasma creatinine were measured with Cobas Pro from Roche Diagnostics (Elecsys Cortisol II and CREP2).

Cortisol binding globulin (CBG) was not measured. Estimated glomerular filtration rate (eGFR) was estimated from plasma creatinine using the revised Lund-Malmö GFR-estimating equation [26]. Analysis of other appetite regulating hormones such as leptin and ghrelin as well as C-peptide could have provided additional value but was not included for financial reasons.

### Self-report measures

A battery of self-report measures was used to assess ED, general psychopathology, and relevant psychological variables.

*The Eating Disorders Inventory-3 (EDI-3)* [27] is a self-report questionnaire with 91 items rated on a 6-point Likert scale. The EDI-3 comprises 12 subscales assessing eating disorder symptoms and related psychological constructs. The first three subscales (Drive for Thinness (DT), Bulimia (B), and Body Dissatisfaction (BD)) assess Eating Disorder Risk. The remaining subscales: Low Self-Esteem (LSE), Personal Alienation (PA), Interpersonal Insecurity (II), Interpersonal Alienation (IA), Interoceptive Deficits (ID), Emotional Dysregulation (ED), Perfectionism (P), Ascetism (A), and Maturity Fears (MF), capture common psychological correlates of eating pathology [19]. The Swedish version of the EDI-3 has been validated in three female samples, i.e. patients with ED (n = 292), psychiatric outpatients (n = 140), and healthy controls (n = 648) [20]. Participant ages ranged from 17 to 50 years. Subscales showed high internal consistency and good test–retest reliability. Also, the instrument with its subscales discriminated well between ED patients and healthy controls, as well as between different ED diagnoses [28]. Cronbach’s *α* for the EDI-3 scales within the present sample ranged from* α* = 0.47 (EDI Bulimia) to *α* = 0.92 (EDI Drive for Thinness), with *α* = 0.66 to *α* = 0.90 for the remaining subscales.

*The Eating Disorder Examination Questionnaire (EDE-Q*) [29, 30] was used to measure ED cognitions and behaviors covering the past 28 days. The EDE-Q measures concern about shape, weight, and eating, as well as eating restraints, and comprises 36 items, scored on a Likert scale from 0–6, and variable were calculated as averages of included items. A higher score indicates more severe ED pathology. The scale has shown high internal consistency and good test–retest reliability in Swedish and international populations [31]. Within the present samples Cronbach’s *α* for the EDE-Q scales were *α* = 0.66 for the total score, Eating Restraint *α* = 0.80, Eating Concern *α* = 0.47, Weight Concern *α* = 0.91, and Shape Concern *α* = 0.96.

*Obsessive–Compulsive Inventory-Revised (OCI-R)* was used to measure Obsessive-compulsory symptoms. The *OCI-R* consists of 18 items scored on a Likert scale ranging from 0 to 4 and has revealed good psychometric properties in international and Swedish populations [31, 32]. The Cronbach’s *α* for the OCI-R within the present sample was* α* = 0.86.

The *Montgomery-Åsberg Depression Rating Scale (MADRS-S)* was used to measure depressive symptoms. The MADRS-S includes nine items, each scored on a Likert scale ranging from 0 to 6, with a maximum score of 63 covering the past three days [33]. The instrument has shown acceptable psychometric properties regarding reliability and validity [34]. Cronbach’s *α* for the MADRS-S within the present sample was* α* = 0.94.

*State-Trait Anxiety Inventory (STAI-S/T)* was used to measure state and trait anxiety. The instrument contains two concepts and consists of two parts with 20 items each, scored on a Likert scale ranging from 1 to 4 and has been adapted in more than 40 languages [35]. The scales have shown good psychometric properties regarding validity and internal consistency in international studies [36]. Cronbach’s *α* within this sample were = 0.95 (STAI-S) and *α* = 0.85 (STAI-T), respectively.

### Ethical considerations

The study was approved by the Swedish ethical review authority: Dnr 2019-06350 and dnr 2021-00766 and performed in accordance with the Declaration of Helsinki. All subjects received both written and oral information about the procedure of the study before giving their written informed consent.

### Statistical analysis

Statistical analysis was performed with STATISTICA (version 12, Statsoft®, Tulsa, USA), and *p* < 0.05 were considered statistically significant. The predominant non-normality distributions of the included variables in the analyses are centered to nonparametric test, including multiple Spearman’s rank correlations, and variables are presented in the text as median and range.

Data are merely descriptive, and the *p*-values have, therefore, not been adjusted for multiple tests e.g. Bonferroni’s correction. Differences were analyzed in two steps by using the Mann–Whitney U-test. Firstly, between subjects diagnosed with AN compared to healthy controls, and secondly, between the two AN subgroups, WRAN and non-WRAN. To increase lucidity, we have tabled the variables in both median and range as well as mean values and SD. Questionnaire items and hormone levels, adjusted for BMI, were calculated using ordinary multiple correlation analysis using the rank values. This is an extension of the similar Spearman correlation between two variables, which is identical to the Pearson correlation between the rank values of two variables.

## Results

Clinical characteristics of the subjects with AN (n = 10); five subjects with WRAN (BMI > 18.5 kg/m^2^) and five subjects with non-WRAN (BMI < 18.5 kg/m^2^), and the healthy controls (n = 7) are summarized in Table 1. Median age in all subjects was 30 (18–35) years and did not differ between groups.

**Table 1:** Patient details separated into controls, those with BMI > 18.5 kg/m^2^ (WRAN; weight restored anorexia nervosa) and those with BMI < 18.5 kg/m^2^ (non-WRAN; non-weight restored anorexia nervosa)

	Controls	AN			Total	Differences (p-values)		
		WRAN	non-WRAN	AN-total		*	**	***
N	7	5	5	10	17			
Age								
Mean (SD)	31.8 (3.6)	27.8 (5.7)	27.9 (5.1)	27.8 (5.1)	29.5 (4.8)			
Median (range)	33 (27–35)	30 (18–33)	29 (21–34)	29 (18–34)	30 (18–35)	0.270	0.432	1.000
Body mass index (BMI; kg/m^2^)								
Mean (SD)	26.9 (5.7)	20.9 (1.8)	16.9 (1.1)	18.9 (2.6)	22.2 (5.7)			
Median (range)	25 (22–36)	21 (19–23)	17 (16–18)	18 (16–23)	22 (16–36)	** < 0.001**	**0.003**	**0.008**
Duration from diagnosis (years)								
Mean (SD)	–	10.0 (2.1)	14.4 (5.5)	12.2 (4.6)	-			
Median (range)	–	9.0 (7.7–12.7)	14.4 (6.8–21.8)	12.2 (6.8–21.8)	-	–	–	**0.222**
Duration amenorrhea (months)								
Mean (SD)	–	0.0 (0.0)	98 (94)	49 (81)	-			
Median (range)	–	0 (0–0)	118 (0–197)	0.0 (0–197)	-	–	–	**0.151**
Serum GLP-1 (pg/mL)								
Mean (SD)	16.7 (2.1)	17.1 (2.0)	37.5 (27.8)	27.3 (21.5)	23.0 (17.0)			
Median (range)	16 (15–21)	17 (15–20)	29 (15–85)	19 (15–85)	18 (15–85)	0.161	**0.048**	0.095
Serum Glucagon (pg/mL)								
Mean (SD)	26.7 (9.3)	38.2 (38.7)	83.8 (115.5)	61.0 (84.7)	46.9 (66.1)			
Median (range)	26 (17–45)	21 (18–107)	34 (26–290)	26 (18–290)	26 (17–290)	0.601	0.202	0.095
Serum GIP (pmol/L)								
Mean (SD)	15.0 (20.2)	58.5 (72.5)	63.0 (59.4)	60.7 (62.5)	41.9 (53.8)			
Median (range)	5 (2–58)	39 (2–182)	37 (14–163)	38 (2–182)	21 (2–182)	0.070	**0.048**	0.841
Serum PYY (ng/mL)								
Mean (SD)	0.57 (0.44)	0.46 (0.35)	0.81 (0.30)	0.64 (0.36)	0.61 (0.38)			
Median (range)	0.4 (0.2–1.5)	0.3 (0.3–1.1)	0.7 (0.6–1.3)	0.6 (0.3–1.3)	0.5 (0.2–1.5)	0.601	0.149	0.095

Differences analysed using non-parametric Mann–Whitney’s U-test. *) Controls vs AN (anorexia nervosa)-total, **) Controls vs AN non-WRAN, ***) WRAN vs AN non-WRAN. P < 0.05 are bolded

BMI was higher in healthy controls (25 (22–35)) compared to all patients with AN (18 (16–23); *p* < 0.001) and BMI was, due to study design, higher in subjects with WRAN (21 (19–23) kg/m^2^) than in non-WRAN subjects (17 (16–18) kg/m^2^; *p* = 0.008). All subjects were non-smokers. One subject in the group of WRAN medicated with a psychotropic drug, quetiapine (50 mg as needed bedtime for mild anxiety symptoms), and four subjects with WRAN were on estrogen/progesterone, and 6 of the controls were either on similar birth control pills (n = 4) or had a contraceptive implant (n = 1), or a hormonal IUD (n = 1). Two subjects in the group of non-WRAN were menstruating irregularly and three were amenorrheic. One subject with non-WRAN after inclusion in the study admitted to occasional purging (about 3 times/week).

**Fig. 1:**
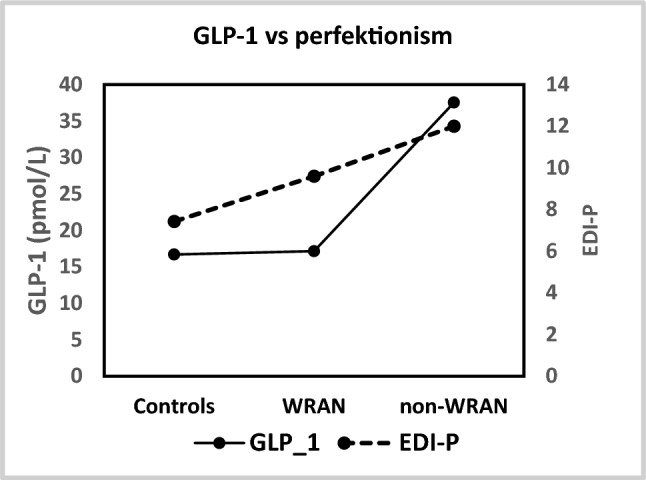
The figure shows a comparison between mean values of serum GLP-1 concentrations respectively mean values for Questionnaire item EDI Perfectionism (EDI-P) for healthy controls (C), those with BMI ≥ 18.5 kg/m^2^ (WRAN; weight restored anorexia nervosa) and those with BMI < 18.5 kg/m^2^ (non-WRAN; non-weight restored anorexia nervosa). Mean value (SD) of serum GLP-1 concentrations for C 16.7 ± 2.1, for WRAN 17.1 ± 2.0 and for non-WRAN 37.5 ± 27.8. Mean value (SD) for EDI-P for C 7.4 ± 3.6, for WRAN 9.6 ± 3.8 and for non-WRAN 12 ± 6.5. p-value for C vs non-WRAN for GLP-1 was 0.048 and for EDI-P 0.202

B-hemoglobin levels were higher in controls (17 (16–18) g/l) than in all subjects diagnosed with AN (129 (113–150), *p* < 0.027). There were no differences between groups in blood pressure or in levels of B-leukocytes, P-ALAT, P-T3, P-T, P-Creatinine or eGFR. No differences between controls and subjects diagnosed with AN, nor between subjects with WRAN and non-WRAN, were found in the levels of the appetite regulating hormones. Serum levels of GLP-1 were, however, higher in subjects with non-WRAN than in controls (29 (15–85) vs 16 (15–21) pg/mL; *p* = 0.048). Also, levels of serum GIP were higher in subjects with non-WRAN compared to controls (37 (14–163) vs 5 (2–58) pmol/L; *p* = 0.048) (15.0 ± 20.2 pmol/L; *p* = 0.048). For further details see Table 1, Supplemental Table 1 and Fig. 1. Also see Supplemental Table 3 for individual data for each participant.

### Correlations between appetite regulating hormones

Serum levels of GLP-1 correlated with serum levels of GIP (r = 0.522; *p* = 0.031) and serum levels of PYY (r = 0.489; *p* = 0.046), while serum levels of PYY correlated with serum levels of GIP (r = 0.523; *p* = 0.031). There were no correlations found between plasma levels of cortisol and the assessed appetite-regulating hormones.

### Psychiatric self-assessment scales

Overall, subjects diagnosed with AN (the AN-total group; n = 10) generally scored higher (i.e. indicated poorer psychological well-being) on the psychiatric self-assessment scales compared with healthy controls (n = 7) (See Table 2 and Supplemental Table 2). The AN-total group scored significantly higher on the EDI total score compared with healthy controls (*Mdn* = 131 vs 51, *p* = 0.002), and also on most subscales, except for the EDI *Bulimia* subscale (*Mdn* = 1 vs 1, *p* = 0.887). The following subscales were elevated in the AN-total group but did not reach statistical significance: EDI *Interpersonal Insecurity* (*Mdn* = 9 vs 7, *p* = 0.133), EDI *Interpersonal Alienation* (*Mdn* = 10 vs 5, *p* = 0.055), EDI *Perfectionism* (*Mdn* = 11 vs 6, *p* = 0.161; see Fig. 1), and EDI *Maturity Fears* (*Mdn* = 11 vs 8, *p* = 0.133). The AN-total group also scored significantly higher on the EDEQ total score (*Mdn* = 3.8 vs 0.5, *p* = 0.003) and on all four subscales: Eating Restraint (*Mdn* = 4.0 vs 0.3, *p* = 0.007), Eating Concern (*Mdn* = 2.4 vs 0.1,*p* = 0.011), Shape Concern (*Mdn* = 4.1 vs 1.4, *p* = 0.005), and Weight Concern (*Mdn* = 3.5 vs 0.4, *p* = 0.007). They also scored higher on the MADRS-S (*Mdn* = 20 vs 6, *p* = 0.005), OCI-R (*Mdn* = 24 vs 10, *p* = 0.010), STAI-S *(Mdn* = 49 vs 34, *p* = 0.023), and STAI-T (*Mdn* = 51 vs 36, *p* = 0.019).

**Table 2:** Patient details separated into controls, those with BMI > 18.5 kg/m^2^ (WRAN; weight restored anorexia nervosa)) and those with BMI < 18.5 kg/m^2^ (non-WRAN; non-weight restored anorexia nervosa)

	Controls	AN			Total	Differences (p-values)		
		WRAN	non-WRAN	AN-total		*	**	***
N	7	5	5	10	17			
EDI-Tot								
Mean (SD)	57.0 (31.2)	142.0 (22.4)	111.4 (40.0)	126.7 (34.6)	98.0 (47.8)			
Median (range)	51 (17–114)	145 (114–173)	118 (49–155)	131 (49–173)	114 (17–173)	**0.002**	**0.048**	0.310
EDI-ED Risk								
Mean (SD)	15.0 (11.5)	51.6 (9.6)	30.2 (18.8)	40.9 (18.0)	30.2 (20.1)			
Median (range)	10 (6–38)	54 (40–65)	29 (12–56)	43 (12–65)	29 (6–65)	**0.005**	0.106	0.151
EDI-Psych								
Mean (SD)	42.0 (21.4)	90.4 (18.5)	81.2 (26.8)	85.8 (22.2)	67.8 (30.7)			
Median (range)	38 (11–76)	96 (60–108)	89 (37–104)	93 (37–108)	76 (11–108)	**0.005**	**0.048**	0.690
EDI-P								
Mean (SD)	7.4 (3.6)	9.6 (3.8)	12.0 (6.5)	10.8 (5.2)	9.4 (4.8)			
Median (range)	6 (3–13)	8 (6–15)	12 (3–21)	11 (3–21)	9 (3–21)	0.161	0.202	0.548
MADRS-S								
Mean (SD)	5.8 (5.3)	18.8 (12.2)	20.2 (8.3)	19.5 (9.8)	14.4 (10.7)			
Median (range)	6 (0–15)	12 (8–33)	21 (7–29)	20 (7–33)	11 (0–33)	**0.005**	**0.017**	1.000
OCI-R								
Mean (SD)	10.6 (8.2)	22.0 (6.0)	25.8 (12.8)	23.9 (9.6)	18.4 (11.1)			
Median (range)	10 (2–23)	23 (12–28)	25 (8–43)	24 (8–43)	22 (2–43)	**0.010**	**0.048**	0.690
STAI-S								
Mean (SD)	34.3 (11.3)	52.5 (11.1)	50.2 (15.0)	51.2 (12.7)	43.8 (14.6)			
Median (range)	34 (23–57)	48 (45–69)	53 (28–67)	49 (28–69)	45 (23–69)	**0.023**	0.106	0.905
STAI-T								
Mean (SD)	39.4 (9.2)	53.4 (10.2)	49.8 (10.8)	51.6 (10.1)	46.6 (11.2)			
Median (range)	36 (28–55)	49 (46–71)	53 (32–58)	51 (32–71)	48 (28–71)	**0.019**	0.106	1.000
EDEQ-Tot								
Mean (SD)	0.8 (0.9)	4.2 (0.5)	2.3 (1.7)	3.3 (1.5)	2.3 (1.8)			
Median (range)	0.5 (0.0–2.5)	4.1 (3.6–5.0)	2.1 (0.7–4.4)	3.8 (0.7–5.0)	2.3 (0.0–5.0)	**0.003**	0.052	0.151

Differences analysed using non-parametric Mann–Whitney’s U-test. *) Controls vs AN (anorexia nervosa)-total, **) Controls vs AN non-WRAN, ***) WRAN vs non-WRAN. p < 0.05 are bolded

When comparing weight recovered subjects (AN non-WRAN; n = 5) and healthy controls (n = 7), the former group demonstrated a significantly higher EDI total score (*Mdn* = 118 vs 51, *p* = 0.048), as well as higher scores on the EDI subscales measuring psychological correlates of eating pathology (*EDI-Psych; Mdn* = 89 vs 38, *p* = 0.048), EDI *Low Self-Esteem* (*Mdn* = 9 vs 3, *p* = 0.030), EDI *Personal Alienation* (*Mdn* = 10 vs 2, *p* = 0.048), and EDI *Ascetism* (*Mdn* = 5 vs 1, *p* = 0.018). They also scored higher on the MADRS-S (*Mdn* = 21 vs 6, *p* = 0.017) and the OCI-R (*Mdn* = 25 vs 10, *p* = 0.048). No significant group differences were observed for the EDEQ or the STAT-S/T. The WRAN group (n = 5) showed significantly higher scores on the EDEQ *Eating Concern* subscale compared with the non-WRAN group (n = 5) (*Mdn* = 2.4 vs 1.0, *p* = 0.016).

For individual data for each participant see Supplemental Table 3 and for correlations between psychiatric self-assessment scales, see Table 3.

**Table 3:** Spearman's rank correlations between self-report measures in the entire group of study participants (n = 17)

Variable	EDI_Tot	EDI_ED_Risk	EDI_Psych	MADRS_S	OCI_R	STAI_S	STAI_T	EDEQ_Tot	EDI_DT	EDI_B
EDI_Tot	1.000	**0.931**	**0.939**	**0.900**	**0.770**	**0.812**	**0.825**	**0.924**	**0.907**	0.342
EDI_ED_Risk		1.000	**0.773**	**0.794**	**0.653**	**0.693**	**0.700**	**0.937**	**0.962**	0.327
EDI_Psych			1.000	**0.942**	**0.845**	**0.884**	**0.905**	**0.797**	**0.757**	0.261
MADRS_S				1.000	**0.852**	**0.849**	**0.878**	**0.777**	**0.744**	0.152
OCI_R					1.000	**0.761**	**0.803**	**0.615**	**0.609**	− 0.042
STAI_S						1.000	**0.977**	**0.679**	**0.660**	0.199
STAI_T							1.000	**0.684**	**0.681**	0.200
EDEQ_Tot								1.000	**0.904**	0.297
EDEQ_R									1.000	0.216
EDEQ_EC										1.000
EDEQ_SC										
EDEQ_WC										

Variable	**EDI_BD**	**EDI_LSE**	**EDI_PA**	**EDI_II**	**EDI_IA**	**EDI_ID**	**EDI_ED**	**EDI_P**	**EDI_A**	**EDI_MF**
EDI_Tot	**0.835**	**0.830**	**0.811**	**0.763**	**0.667**	**0.782**	**0.706**	0.329	**0.958**	**0.712**
EDI_ED Risk	**0.939**	**0.819**	**0.663**	**0.623**	**0.530**	**0.662**	**0.654**	0.163	**0.883**	**0.576**
EDI_Psych	**0.653**	**0.736**	**0.868**	**0.760**	**0.703**	**0.844**	**0.641**	**0.514**	**0.930**	**0.724**
MADRS_S	**0.651**	**0.702**	**0.777**	**0.710**	**0.667**	**0.846**	**0.601**	**0.537**	**0.910**	**0.711**
OCI_R	**0.530**	**0.575**	**0.744**	**0.596**	**0.636**	**0.716**	0.465	**0.552**	**0.803**	**0.599**
STAI_S	**0.636**	**0.627**	**0.905**	**0.830**	**0.715**	**0.806**	0.373	0.435	**0.807**	**0.675**
STAI_T	**0.636**	**0.657**	**0.911**	**0.746**	**0.762**	**0.825**	0.424	0.465	**0.822**	**0.673**
EDEQ_Tot	**0.903**	**0.879**	**0.703**	**0.622**	0.403	**0.582**	**0.592**	0.256	**0.901**	**0.498**
EDI_DT	**0.874**	**0.904**	**0.662**	**0.624**	0.467	**0.556**	**0.651**	0.115	**0.830**	**0.649**
EDI_B	0.335	0.065	0.067	0.303	0.368	**0.491**	0.400	− 0.189	0.342	0.120
EDI_BD	1.000	**0.751**	**0.598**	**0.540**	**0.506**	**0.589**	**0.489**	0.032	**0.830**	0.380
EDI_LSE		1.000	**0.705**	**0.595**	0.364	0.411	0.443	0.275	**0.757**	**0.601**
EDI_PA			1.000	**0.780**	**0.728**	**0.711**	0.392	0.464	**0.748**	**0.701**
EDI_II				1.000	**0.664**	**0.603**	0.388	0.243	**0.666**	**0.853**
EDI_IA					1.000	**0.833**	**0.506**	0.143	**0.660**	**0.580**
EDI_ID						1.000	**0.565**	0.356	**0.802**	**0.518**
EDI_ED							1.000	0.059	**0.688**	**0.558**
EDI_P								1.000	0.344	0.302
EDI_A									1.000	**0.590**
EDI_MF										1.000

EDI: Eating Disorder Inventory-3; ED: Risk Eating Disorder Risk Composite, EDI: Psych Psychological Maladjustment Composite; MADRS-S: Montgomery-Åsberg Depression Rating Scale Self-Assessment; OCI-R: Obsessive–Compulsive-Inventory-Revised; STAI: State-Trait Anxiety Inventory; EDEQ: Eating Disorders Examination Questionnaire; EDEQ-R

### Correlations between appetite regulating hormones and psychiatric self-assessment scales

GLP-1 was correlated with ED *Perfectionism* (*r* = 0.749, *p* = 0.001). When adjusted for BMI the correlation remained (*r* = 0.768, *p* = 0.001). No other correlations were found, but adjustments for BMI were also made for associations with a p < 0.10. Serum levels of GIP were, previous to adjustment för BMI, not correlated to EDI *Perfectionism* (*r* = 0.421; *p* = 0.092), which remained after adjustment (*r* = 0.333; *p* = 0.284), nor did levels of GIP correlate with OCI-R (*r* = 0.452; *p* = 0.059), which also remained after adjustment BMI (*r* = 0.394; *p* = 0.118).

### Subjects with a psychotropic drug, respectively purging

Since psychotropic drugs and purging may influence levels of appetite-regulating hormones we further analyzed our main findings in relation to these two patients. This analysis showed that neither excluding the subject with the small dose of quetiapine medication nor the subject with purging weakened the association between GLP-1 and perfection (*r* = 0.81, *p* < 0.0001 and *r* = 0.79, *p* < 0.001, respectively). The subject that was on quetiapine belonged to the group of WRAN, and, thus, did not influence the comparison between basal serum levels of GLP-1 levels in subjects with non-WRAN and controls. If the subject with purging was omitted GLP-1 was no longer significantly higher in the group of non-WRAN subjects (*p* = 0.12) due to the small number of patients, but not due to the subject´s serum GLP-1 value, which was close to the median (37.44 vs 37.54 pg/mL), and the subject was therefore not excluded from the study.

## Discussion

The two main findings of the study were that GLP-1 levels were, independent of BMI, associated with perfectionism, and that fasting serum levels of GLP-1 and GIP were elevated in subjects with non-WRAN (BMI < 18.5 kg/m^2^) compared to controls.

The small or non-existent differences in routine laboratory test results, and in levels of appetite-regulating hormones between groups, may reflect how subjects with non-WRAN had been ill for many years having adapted to semi-starvation in accordance with moderately low BMIs. For instance, a higher morning cortisol value would have been expected in the group of subjects with non-WRAN [37], as well as correlations between cortisol levels and self-assessment scores, and also higher levels of PYY in both subjects with non-WRAN and WRAN [38], as well as lower levels of thyroid hormone levels in the non-WRAN group underscoring this point [10]. Although not significantly, cortisol levels were actually slightly higher in the group with non-WRAN than WRAN, when the opposite was expected. This could be explained by a higher use of estrogens in the former group, raising total cortisol levels by increasing levels of CBG. However, our findings are in agreement with those presented in a study by Lawson et al. [13].

To our knowledge, there is only one published study assessing GLP-1 levels in adults with AN showing higher basal GLP-1 levels in comparison to constitutionally lean, although the circadian GLP-1 tended to be higher in the AN group than in the control subjects [39]. Heruc et al. reported that adolescents with AN exhibited elevated fasting plasma levels of GLP-1 compared to healthy controls. However, the difference disappeared after accounting for the higher basal levels of the meal response (incremental AUC) [40]. Contradictory results were found in a study by Tomasik and co-workers in young girls (13 – 16 years) with AN [14]. Levels of GIP were, in the same study, elevated both basally and postprandially, but in a previous study [41] only postprandially, and these results were not in agreement with two other studies [40, 42]. In the study by Kolb and colleagues [16], fasting levels of GIP, but not GLP-1, were elevated. Overall, studies examining GLP-1 and GIP in anorexia nervosa (AN) remain limited and have not yet reached a consensus [37]. In the aforementioned study by Heruc et al., glucagon levels were higher in patients with AN [34], in contrast to the findings of both our study and that of Kolb et al. [16].

As to the psychiatric self-assessment scales, multiple differences between subjects previously diagnosed with AN and HC were noted, with AN generally being accompanied by poorer psychological well-being. Other studies have demonstrated an association between leptin levels and decreased depressive symptoms, independent of body fat or weight [42]. Furthermore, cortisol, PYY and leptin were, in another study by the same research group, associated with eating pathologies such as restraint, eating concerns, and body image disturbances, and these correlations remained significant for cortisol and PYY but not for leptin, probably reflecting leptin´s role as a marker for fat rather than a possible mediator of disordered eating thoughts and behavior (13). In the present study serum levels of GIP were elevated in the non-WRAN group compared to controls. This finding, as the finding of increased levels of GLP-1 in the same comparison, must be interpreted with caution given the small number of subjects in each group. Serum levels of GIP were not correlated with obsessiveness (OCI-R; *p* = 0.118 after adjusting for BMI), but because of the small sample size this finding may be of interest and stimulate further studies in patients with AN, obsessiveness being a common trait in AN [43].

Within the examined sample, the association between GLP-1 and perfectionism remained significant after adjustment for BMI, suggesting that this relationship is not merely mediated by low body weight. This finding suggests a potential association between incretins and psychopathologies in AN, similar to what has been observed with other appetite regulating hormones. Of note, perfectionism is regarded as a central maintaining factor in eating disorders [44] and studies have indicated a correlation between elevated perfectionism and more severe eating disorder symptoms [45]. Perfectionism is hypothesized to not only play a pivotal role in the development of eating disorders but also to contribute to their sustainment [46]. Furthermore, the onset of AN has been linked to an increase in perfectionistic tendencies [47]. Most research suggests that perfectionism remains elevated even after recovery, indicating that it is more likely a trait than a state-dependent characteristic [48], and perfectionism has been associated with higher ED symptoms and lower remission in adolescents with ED [49]. If GLP-1 in patients with anorexia nervosa (AN) mediates both heightened perfectionism and an early sensation of satiety, one would expect not only elevated GLP-1 levels in the non-weight-restored AN (non-WRAN) group, but also higher levels of perfectionism in this group. However, no such difference was observed (p = 0.202). This may reflect limited statistical power due to the small sample size, or it may indicate that early satiety in AN is mediated by other mechanisms. Alternatively, the absence of an observed difference may be explained by the fact that perfectionism as measured by the EDI 3 reflects state rather than trait-dependent perfectionism. Assuming the observed association between GLP-1 levels and perfectionism in AN hold true, and that GLP-1 levels are subsequently confirmed to be elevated both at baseline and postprandially in this population, it remains to be determined whether GLP-1 contributes to the heightened perfectionism and early satiety reported in individuals with AN—factors that may hinder recovery. Notably, Kolb et al. did not examine EDI subscales, limiting direct comparison with our findings [16]. The study by Kolb and colleagues included a larger cohort (n = 80); however, in contrast to our sample, participants were substantially younger (mean age 14.9 vs 27.9 years) and diagnosed with acute rather than chronic AN. These differences in age and illness stage may partly explain the discrepant findings regarding GLP-1 levels. To the best of our knowledge this is the first study to demonstrate an association between serum levels of GLP-1 and perfectionism. These findings are particularly intriguing given the documented metabolic and possible psychological effects of incretin-based medications in obesity treatment. However, given the small sample size, these findings should be interpreted with care, and further studies are required to confirm these results.

## Strength and limits

A strength of the study was the thorough evaluation of psychiatric symptoms using validated assessment scales, although several limitations need to be addressed. The cross-sectional design of this study does not allow us to draw conclusions regarding causality. In addition, the number of included patients and controls were small, and, therefore, actual associations between psychiatric symptoms and hormone levels, as well as differences in levels of appetite-regulating hormones between groups (type II error), may have been missed. Contrary to previous results, perfectionism [8] was not higher in the group of subjects formerly diagnosed with AN in the present study compared to controls. Findings from this study merit further research in a larger population with pronounced symptoms, preferably also investigating levels of incretins after a standardized meal, encompassing individuals with both acute and chronic anorexia nervosa (AN), as well as patients at different stages of weight restoration, including those with recent and long-term recovery.

Furthermore, the study included weight-dependent subgroups of AN, i.e. comprising women who had been diagnosed with AN who had achieved weight restoration (WRAN) and those with non-restored weight (non-WRAN; BMI < 18.5 kg/m^2^). This may be debatable since criteria for cured AN can differ between treatment centers. However, appetite regulating hormones [50, 51] and psychopathologies [8, 52] are not necessarily normalized in patients with AN, although weight has been restored, which may question when AN should be considered cured. All subjects previously diagnosed with AN should have undergone a Structured Interview for DSM-IV Disorders to refine the group of subjects with non-WRAN and the group of subjects with WRAN, which they had not. Despite the limitations of the study, a strong association between GLP-1 levels and perfectionism was revealed, independent of BMI. This finding seems unlikely to be a random finding and was consistent regardless of how subjects previously diagnosed with AN were subdivided.

In conclusion, in a mixed group of individuals diagnosed with AN and matched controls, GLP-1 levels were elevated in subjects with non-WRAN and also independently of BMI associated with perfectionism, a typical characteristic of AN. Elevated GLP-1 levels may perpetuate AN symptoms, emphasizing the need for further research into their role in the metabolic and psychiatric dimensions of AN.

## What is already known on this subject?

Findings from incretin-based treatments for obesity may have implications for AN. Although previous studies on incretins in AN have yielded mixed results, emerging evidence is beginning to clarify the role of incretin hormones in this disorder.

## What this study adds?

This study preformed in a mixed group of individuals diagnosed with AN and matched controls presents a novel finding of an association between serum levels of GLP-1 and perfectionism, indicating a possible role for GLP-1 in AN and warrant further research.

## Competing interests

The authors declare no competing interests.

## Supplementary Information

Below is the link to the electronic supplementary material.

Supplementary Material 1.

## Data Availability

The datasets generated during and/or analysed during the current study are available from the corresponding author on reasonable request.

## Abbreviations

AN: Anorexia nervosa
HC: Healthy controls
WRAN: Weight restored anorexia nervosa
OR: Odds ratio
CI: Confidence Interval
