# Supplementary material for: GLP-1 is associated with perfectionism in Swedish women with anorexia nervosa, independent of BMI
Source: Eat Weight Disord. 2026 Mar 3;31(1):25. doi: 10.1007/s40519-026-01831-x (PMC13061841; doi:10.1007/s40519-026-01831-x)
Supplement: Supplementary file 1 — Supplementary Material 1. [file 40519_2026_1831_MOESM1_ESM.docx]

**Supplemental tables**

**Supplemental table 1.** Patient details separated into controls, those with BMI>18 (WRAN; weight restored anorexia nervosa) and those with BMI<18 (non-WRAN; non-weight restored anorexia nervosa). Differences analysed using non-parametric Mann-Whitney’s U-test. *) Controls vs AN (anorexia nervosa)-total, **) Controls vs AN non-WRAN, ***) WRAN vs AN non-WRAN. P-values < 0.05 are bolded.

|  |  | **Controls** |  | **AN** | | | |  | **Total** |  |  | **Differences (p-values)** |  |
| --- | --- | --- | --- | --- | --- | --- | --- | --- | --- | --- | --- | --- | --- |
|  |  |  |  | **WRAN** |  | **non-WRAN** | **AN-total** |  |  |  | ***** | ****** | ******* |
| **N** |  | 7 |  | 5 |  | 5 | 10 |  | 17 |  |  |  |  |
| **Age** | |  |  |  |  |  |  |  |  |  |  |  |  |
|  | Mean (SD) | 31.8 (3.6) |  | 27.8 (5.7) |  | 27.9 (5.1) | 27.8 (5.1) |  | 29.5 (4.8) |  |  |  |  |
|  | Median (range) | 33 (27-35) |  | 30 (18-33) |  | 29 (21-34) | 29 (18-34) |  | 30 (18-35) |  | 0.270 | 0.432 | 1.000 |
| **Height (cm)** | |  |  |  |  |  |  |  |  |  |  |  |  |
|  | Mean (SD) | 167.1 (7.4) |  | 163.8 (4.3) |  | 160.2 (5.9) | 162.0 (5.2) |  | 164.1 (6.5) |  |  |  |  |
|  | Median (range) | 166 (156-180) |  | 161 (160-169) |  | 161 (152-167) | 161 (152-169) |  | 164 (152-180) |  | 0.133 | 0.149 | 0.421 |
| **Weight (kg)** | |  |  |  |  |  |  |  |  |  |  |  |  |
|  | Mean (SD) | 74.3 (11.3) |  | 56.0 (2.5) |  | 43.3 (3.8) | 49.7 (7.3) |  | 59.8 (15.3) |  |  |  |  |
|  | Median (range) | 72 (61-95) |  | 55 (54-60) |  | 42 (41-50) | 52 (41-60) |  | 57 (41-95) |  | **<0.001** | **0.003** | **0.008** |
| **Body mass index (BMI; kg/m^2^)** | | |  |  |  |  |  |  |  |  |  |  |  |
|  | Mean (SD) | 26.9 (5.7) |  | 20.9 (1.8) |  | 16.9 (1.1) | 18.9 (2.6) |  | 22.2 (5.7) |  |  |  |  |
|  | Median (range) | 25 (22-36) |  | 21 (19-23) |  | 17 (16-18) | 18 (16-23) |  | 22 (16-36) |  | **<0.001** | **0.003** | **0.008** |
| **Duration from diagnosis (yrs)** | | |  |  |  |  |  |  |  |  |  |  |  |
|  | Mean (SD) | - |  | 10.0 (2.1) |  | 14.4 (5.5) | 12.2 (4.6) |  | - |  |  |  |  |
|  | Median (range) | - |  | 9.0 (7.7-12.7) |  | 14.4 (6.8-21.8) | 12.2 (6.8-21.8) |  | - |  | **-** | **-** | **0.222** |
| **Duration amenorrhea (months)** | | |  |  |  |  |  |  |  |  |  |  |  |
|  | Mean (SD) | - |  | 0.0 (0.0) |  | 98 (94) | 49 (81) |  | - |  |  |  |  |
|  | Median (range) | - |  | 0 (0-0) |  | 118 (0-197) | 0.0 (0-197) |  | - |  | **-** | **-** | **0.151** |
| **Systolic blood pressure (mmHg)** | | |  |  |  |  |  |  |  |  |  |  |  |
|  | Mean (SD) | 117.5 (7.6) |  | 108.6 (18.4) |  | 101.8 (19.5) | 105.2 (18.2) |  | 109.8 (16.0) |  |  |  |  |
|  | Median (range) | 115 (111-130) |  | 110 (88-134) |  | 105 (79-125) | 108 (79-134) |  | 113 (79-134) |  | 0.220 | 0.247 | 0.548 |
| **Blood-hemoglobin (g/L)** | | |  |  |  |  |  |  |  |  |  |  |  |
|  | Mean (SD) | 137.4 (8.2) |  | 128.0 (13.3) |  | 128.0 (8.9) | 128.0 (10.7) |  | 131.9 (10.6) |  |  |  |  |
|  | Median (range) | 136 (128-153) |  | 124 (116-150) |  | 130 (113-135) | 129 (113-150) |  | 131 (113-153) |  | **0.043** | 0.106 | 0.690 |
| **Blood leucocytes (10 E9/L)** | | |  |  |  |  |  |  |  |  |  |  |  |
|  | Mean (SD) | 5.8 (2.1) |  | 4.9 (0.7) |  | 5.9 (2.1) | 5.4 (1.5) |  | 5.6 (1.7) |  |  |  |  |
|  | Median (range) | 5.3 (3.6-8.6) |  | 5.3 (3.9-5.4) |  | 5.9 (2.7-8.0) | 5.4 (2.7-8.0) |  | 5.4 (2.7-8.6) |  | 0.887 | 1.000 | 0.222 |
| **Plasma-ALAT (ukat/L)** | | |  |  |  |  |  |  |  |  |  |  |  |
|  | Mean (SD) | 0.30 (0.12) |  | 0.30 (0.12) |  | 0.79 (0.60) | 0.55 (0.48) |  | 0.45 (0.39) |  |  |  |  |
|  | Median (range) | 0.2 (0.2-0.5) |  | 0.3 (0.2-0.4) |  | 0.7 (0.2-1.6) | 0.4 (0.2-1.6) |  | 0.4 (0.2-1.6) |  | 0.435 | 0.286 | 0.200 |
| **TSH (Thyroid-stimulating hormone)** | | |  |  |  |  |  |  |  |  |  |  |  |
|  | Mean (SD) | 1.80 (0.91) |  | 2.08 (0.87) |  | 1.03 (0.57) | 1.61 (0.90) |  | 1.69 (0.88) |  |  |  |  |
|  | Median (range) | 1.9 (0.5-3.3) |  | 2.3 (0.9-3.2) |  | 0.9 (0.5-1.8) | 1.6 (0.5-3.2) |  | 1.8 (0.5-3.3) |  | 0.758 | 0.164 | 0.111 |
| **Free plasma triiodothyronine (t3; pmol/L)** | | | | |  |  |  |  |  |  |  |  |  |
|  | Mean (SD) | 4.83 (0.66) |  | 4.44 (0.84) |  | 3.94 (1.15) | 4.19 (0.98) |  | 4.43 (0.91) |  |  |  |  |
|  | Median (range) | 4.6 (4.2-6.0) |  | 4.6 (3.5-5.5) |  | 4.2 (2.8-5.5) | 4.3 (2.8-5.5) |  | 4.5 (2.8-6.0) |  | 0.263 | 0.126 | 0.421 |
| **Free plasma tyroxin (pmol/L)** | | |  |  |  |  |  |  |  |  |  |  |  |
|  | Mean (SD) | 15.0 (1.2) |  | 14.4 (3.8) |  | 15.2 (2.8) | 14.8 (3.2) |  | 14.9 (2.5) |  |  |  |  |
|  | Median (range) | 15 (14-17) |  | 15 (10-18) |  | 16 (12-19) | 16 (10-19) |  | 15 (10-19) |  | 0.962 | 1.000 | 0.690 |
| **Plasma creatinine (umol/L)** | | |  |  |  |  |  |  |  |  |  |  |  |
|  | Mean (SD) | 72.14 (7.29) |  | 66.40 (10.97) |  | 65.40 (16.80) | 65.90 (13.39) |  | 68.47 (11.44) |  |  |  |  |
|  | Median (range) | 75 (58-78) |  | 64 (56-83) |  | 60 (43-85) | 62 (43-85) |  | 71 (43-85) |  | 0.475 | 0.876 | 1.000 |
| **Plasma estimated GFR (mL/min/m2)** | | | |  |  |  |  |  |  |  |  |  |  |
|  | Mean (SD) | 84.3 (4.6) |  | 86.2 (7.4) |  | 84.6 (7.6) | 85.4 (7.1) |  | 84.9 (6.1) |  |  |  |  |
|  | Median (range) | 84 (79-90) |  | 90 (73-90) |  | 90 (74-90) | 90 (73-90) |  | 88 (73-90) |  | 0.536 | 0.876 | 1.000 |
| **Plasma cortisol (nmol/L)** | | |  |  |  |  |  |  |  |  |  |  |  |
|  | Mean (SD) | 337 (307) |  | 402 (130) |  | 387 (285) | 394 (209) |  | 371 (246) |  |  |  |  |
|  | Median (range) | 220 (76-980) |  | 440 (260-580) |  | 380 (94-730) | 410 (94-730) |  | 300 (76-980) |  | 0.417 | 0.876 | 1.000 |
| **Serum GLP-1 (pg/mL)** | | |  |  |  |  |  |  |  |  |  |  |  |
|  | Mean (SD) | 16.7 (2.1) |  | 17.1 (2.0) |  | 37.5 (27.8) | 27.3 (21.5) |  | 23.0 (17.0) |  |  |  |  |
|  | Median (range) | 16 (15-21) |  | 17 (15-20) |  | 29 (15-85) | 19 (15-85) |  | 18 (15-85) |  | 0.161 | **0.048** | 0.095 |
| **Serum Glucagon (pg/mL)** | | |  |  |  |  |  |  |  |  |  |  |  |
|  | Mean (SD) | 26.7 (9.3) |  | 38.2 (38.7) |  | 83.8 (115.5) | 61.0 (84.7) |  | 46.9 (66.1) |  |  |  |  |
|  | Median (range) | 26 (17-45) |  | 21 (18-107) |  | 34 (26-290) | 26 (18-290) |  | 26 (17-290) |  | 0.601 | 0.202 | 0.095 |
| **Serum GIP (pmol/L)** | | |  |  |  |  |  |  |  |  |  |  |  |
|  | Mean (SD) | 15.0 (20.2) |  | 58.5 (72.5) |  | 63.0 (59.4) | 60.7 (62.5) |  | 41.9 (53.8) |  |  |  |  |
|  | Median (range) | 5 (2-58) |  | 39 (2-182) |  | 37 (14-163) | 38 (2-182) |  | 21 (2-182) |  | 0.070 | **0.048** | 0.841 |
| **Serum PYY ng/mL)** | |  |  |  |  |  |  |  |  |  |  |  |  |
|  | Mean (SD) | 0.57 (0.44) |  | 0.46 (0.35) |  | 0.81 (0.30) | 0.64 (0.36) |  | 0.61 (0.38) |  |  |  |  |
|  | Median (range) | 0.4 (0.2-1.5) |  | 0.3 (0.3-1.1) |  | 0.7 (0.6-1.3) | 0.6 (0.3-1.3) |  | 0.5 (0.2-1.5) |  | 0.601 | 0.149 | 0.095 |

**Supplemental table 2.** Patient details separated into controls, those with BMI>18 (WRAN; weight restored anorexia nervosa)) and those with BMI<18 (non-WRAN; non-weight restored anorexia nervosa). Differences analysed using non-parametric Mann-Whitney’s U-test. *) Controls vs AN (anorexia nervosa)-total, **) Controls vs AN non-WRAN, ***) WRAN vs non-WRAN. p-values < 0.05 are bolded.

|  |  | **Controls** |  | **AN** | | | |  | **Total** |  |  | **Differences (p-values)** |  |
| --- | --- | --- | --- | --- | --- | --- | --- | --- | --- | --- | --- | --- | --- |
|  |  |  |  | **WRAN** |  | **non-WRAN** | **AN-total** |  |  |  | ***** | ****** | ******* |
| **N** |  | 7 |  | 5 |  | 5 | 10 |  | 17 |  |  |  |  |
| **EDI-Tot** |  |  |  |  |  |  |  |  |  |  |  |  |  |
|  | Mean (SD) | 57.0 (31.2) |  | 142.0 (22.4) |  | 111.4 (40.0) | 126.7 (34.6) |  | 98.0 (47.8) |  |  |  |  |
|  | Median (range) | 51 (17-114) |  | 145 (114-173) |  | 118 (49-155) | 131 (49-173) |  | 114 (17-173) |  | **0.002** | **0.048** | 0.310 |
| **EDI-ED Risk** |  |  |  |  |  |  |  |  |  |  |  |  |  |
|  | Mean (SD) | 15.0 (11.5) |  | 51.6 (9.6) |  | 30.2 (18.8) | 40.9 (18.0) |  | 30.2 (20.1) |  |  |  |  |
|  | Median (range) | 10 (6-38) |  | 54 (40-65) |  | 29 (12-56) | 43 (12-65) |  | 29 (6-65) |  | **0.005** | 0.106 | 0.151 |
| **EDI-Psych** |  |  |  |  |  |  |  |  |  |  |  |  |  |
|  | Mean (SD) | 42.0 (21.4) |  | 90.4 (18.5) |  | 81.2 (26.8) | 85.8 (22.2) |  | 67.8 (30.7) |  |  |  |  |
|  | Median (range) | 38 (11-76) |  | 96 (60-108) |  | 89 (37-104) | 93 (37-108) |  | 76 (11-108) |  | **0.005** | **0.048** | 0.690 |
| **EDI-DT** |  |  |  |  |  |  |  |  |  |  |  |  |  |
|  | Mean (SD) | 4.14 (4.22) |  | 19.60 (4.77) |  | 11.80 (9.23) | 15.70 (8.06) |  | 10.94 (8.81) |  |  |  |  |
|  | Median (range) | 3 (0-10) |  | 20 (14-26) |  | 16 (2-22) | 17 (2-26) |  | 10 (0-26) |  | **0.007** | 0.149 | 0.310 |
| **EDI-B** |  |  |  |  |  |  |  |  |  |  |  |  |  |
|  | Mean (SD) | 2.00 (3.16) |  | 5.60 (3.78) |  | 0.20 (0.45) | 2.90 (3.81) |  | 2.53 (3.48) |  |  |  |  |
|  | Median (range) | 1 (0-9) |  | 6 (0-9) |  | 0 (0-1) | 1 (0-9) |  | 1 (0-9) |  | 0.887 | 0.106 | 0.056 |
| **EDI-BD** |  |  |  |  |  |  |  |  |  |  |  |  |  |
|  | Mean (SD) | 8.9 (5.5) |  | 26.4 (7.0) |  | 18.2 (10.2) | 22.3 (9.3) |  | 16.8 (10.3) |  |  |  |  |
|  | Median (range) | 6 (5-19) |  | 25 (18-35) |  | 13 (10-34) | 23 (10-35) |  | 14 (5-35) |  | **0.005** | 0.073 | 0.222 |
| **EDI-LSE** |  |  |  |  |  |  |  |  |  |  |  |  |  |
|  | Mean (SD) | 4.1 (3.3) |  | 12.0 (5.1) |  | 10.4 (4.4) | 11.2 (4.6) |  | 8.3 (5.4) |  |  |  |  |
|  | Median (range) | 3 (0-9) |  | 10 (7-20) |  | 9 (6-16) | 10 (6-20) |  | 7 (0-20) |  | **0.003** | **0.030** | 0.548 |

| **EDI-PA** |  |  |  |  |  |  |  |  |  |  |  |  |  |
| --- | --- | --- | --- | --- | --- | --- | --- | --- | --- | --- | --- | --- | --- |
|  | Mean (SD) | 3.6 (3.2) |  | 8.8 (0.8) |  | 8.8 (4.5) | 8.8 (3.1) |  | 6.6 (4.0) |  |  |  |  |
|  | Median (range) | 2 (1-9) |  | 9 (8-10) |  | 10 (2-14) | 9 (2-14) |  | 8 (1-14) |  | **0.007** | **0.048** | 0.690 |
| **EDI-II** |  |  |  |  |  |  |  |  |  |  |  |  |  |
|  | Mean (SD) | 6.3 (3.4) |  | 9.2 (3.5) |  | 9.4 (4.5) | 9.3 (3.8) |  | 8.1 (3.8) |  |  |  |  |
|  | Median (range) | 7 (2-12) |  | 9 (5-14) |  | 9 (4-16) | 9 (4-16) |  | 7 (2-16) |  | 0.133 | 0.268 | 1.000 |
| **EDI-IA** |  |  |  |  |  |  |  |  |  |  |  |  |  |
|  | Mean (SD) | 5.4 (3.5) |  | 9.0 (3.9) |  | 10.0 (4.1) | 9.5 (3.8) |  | 7.8 (4.1) |  |  |  |  |
|  | Median (range) | 5 (1-12) |  | 9 (4-15) |  | 11 (3-13) | 10 (3-15) |  | 8 (1-15) |  | 0.055 | 0.106 | 0.548 |
| **EDI-ID** |  |  |  |  |  |  |  |  |  |  |  |  |  |
|  | Mean (SD) | 5.0 (4.2) |  | 14.6 (7.4) |  | 10.8 (6.4) | 12.7 (6.8) |  | 9.5 (6.9) |  |  |  |  |
|  | Median (range) | 4 (0-13) |  | 16 (4-23) |  | 11 (1-17) | 14 (1-23) |  | 9 (0-23) |  | **0.033** | 0.149 | 0.421 |
| **EDI-ED** |  |  |  |  |  |  |  |  |  |  |  |  |  |
|  | Mean (SD) | 1.6 (2.1) |  | 7.0 (5.0) |  | 3.8 (4.5) | 5.4 (4.8) |  | 3.8 (4.3) |  |  |  |  |
|  | Median (range) | 1 (0-6) |  | 7 (2-13) |  | 2 (0-10) | 5 (0-13) |  | 2 (0-13) |  | **0.070** | 0.530 | 0.222 |
| **EDI-P** |  |  |  |  |  |  |  |  |  |  |  |  |  |
|  | Mean (SD) | 7.4 (3.6) |  | 9.6 (3.8) |  | 12.0 (6.5) | 10.8 (5.2) |  | 9.4 (4.8) |  |  |  |  |
|  | Median (range) | 6 (3-13) |  | 8 (6-15) |  | 12 (3-21) | 11 (3-21) |  | 9 (3-21) |  | 0.161 | 0.202 | 0.548 |
| **EDI-A** |  |  |  |  |  |  |  |  |  |  |  |  |  |
|  | Mean (SD) | 1.1 (1.5) |  | 8.0 (4.3) |  | 4.8 (2.5) | 6.4 (3.7) |  | 4.2 (4.0) |  |  |  |  |
|  | Median (range) | 1 (0-4) |  | 7 (4-13) |  | 5 (1-7) | 6 (1-13) |  | 4 (0-13) |  | **0.001** | **0.018** | 0.421 |
| **EDI-MF** |  |  |  |  |  |  |  |  |  |  |  |  |  |
|  | Mean (SD) | 7.6 (4.4) |  | 11.2 (4.1) |  | 11.2 (4.2) | 11.2 (3.9) |  | 9.7 (4.4) |  |  |  |  |
|  | Median (range) | 8 (2-14) |  | 11 (8-18) |  | 10 (7-18) | 11 (7-18) |  | 9 (2-18) |  | 0.133 | 0.202 | 1.000 |
| **MADRS-S** |  |  |  |  |  |  |  |  |  |  |  |  |  |
|  | Mean (SD) | 5.8 (5.3) |  | 18.8 (12.2) |  | 20.2 (8.3) | 19.5 (9.8) |  | 14.4 (10.7) |  |  |  |  |
|  | Median (range) | 6 (0-15) |  | 12 (8-33) |  | 21 (7-29) | 20 (7-33) |  | 11 (0-33) |  | **0.005** | **0.017** | 1.000 |

| **OCI-R** |  |  |  |  |  |  |  |  |  |  |  |  |  |
| --- | --- | --- | --- | --- | --- | --- | --- | --- | --- | --- | --- | --- | --- |
|  | Mean (SD) | 10.6 (8.2) |  | 22.0 (6.0) |  | 25.8 (12.8) | 23.9 (9.6) |  | 18.4 (11.1) |  |  |  |  |
|  | Median (range) | 10 (2-23) |  | 23 (12-28) |  | 25 (8-43) | 24 (8-43) |  | 22 (2-43) |  | **0.010** | **0.048** | 0.690 |
| **STAI-S** |  |  |  |  |  |  |  |  |  |  |  |  |  |
|  | Mean (SD) | 34.3 (11.3) |  | 52.5 (11.1) |  | 50.2 (15.0) | 51.2 (12.7) |  | 43.8 (14.6) |  |  |  |  |
|  | Median (range) | 34 (23-57) |  | 48 (45-69) |  | 53 (28-67) | 49 (28-69) |  | 45 (23-69) |  | **0.023** | 0.106 | 0.905 |
| **STAI-T** |  |  |  |  |  |  |  |  |  |  |  |  |  |
|  | Mean (SD) | 39.4 (9.2) |  | 53.4 (10.2) |  | 49.8 (10.8) | 51.6 (10.1) |  | 46.6 (11.2) |  |  |  |  |
|  | Median (range) | 36 (28-55) |  | 49 (46-71) |  | 53 (32-58) | 51 (32-71) |  | 48 (28-71) |  | **0.019** | 0.106 | 1.000 |
| **EDEQ-Tot** |  |  |  |  |  |  |  |  |  |  |  |  |  |
|  | Mean (SD) | 0.8 (0.9) |  | 4.2 (0.5) |  | 2.3 (1.7) | 3.3 (1.5) |  | 2.3 (1.8) |  |  |  |  |
|  | Median (range) | 0.5 (0.0-2.5) |  | 4.1 (3.6-5.0) |  | 2.1 (0.7-4.4) | 3.8 (0.7-5.0) |  | 2.3 (0.0-5.0) |  | **0.003** | 0.052 | 0.151 |
| **EDEQ-R** |  |  |  |  |  |  |  |  |  |  |  |  |  |
|  | Mean (SD) | 0.7 (0.9) |  | 4.4 (1.1) |  | 2.8 (2.3) | 3.6 (1.9) |  | 2.5 (2.1) |  |  |  |  |
|  | Median (range) | 0.3 (0.0-2.0) |  | 4.2 (3.2-6.0) |  | 2.0 (0.0-6.0) | 4.0 (0.0-6.0) |  | 2.0 (0.0-6.0) |  | **0.007** | 0.126 | 0.310 |
| **EDEQ-EC** |  |  |  |  |  |  |  |  |  |  |  |  |  |
|  | Mean (SD) | 0.33 (0.63) |  | 2.96 (0.82) |  | 1.08 (1.03) | 2.02 (1.32) |  | 1.39 (1.37) |  |  |  |  |
|  | Median (range) | 0.1 (0.0-1.6) |  | 2.4 (2.4-4.2) |  | 1.0 (0.0-2.4) | 2.4 (0.0-4.2) |  | 1.3 (0.0-4.2) |  | **0.011** | 0.177 | **0.016** |
| **EDEQ-SC** |  |  |  |  |  |  |  |  |  |  |  |  |  |
|  | Mean (SD) | 1.3 (1.2) |  | 4.9 (1.0) |  | 3.0 (1.9) | 3.9 (1.8) |  | 2.9 (2.0) |  |  |  |  |
|  | Median (range) | 1.4 (0.0-3.3) |  | 5.4 (3.6-6.0) |  | 3.4 (0.3-5.3) | 4.1 (0.3-6.0) |  | 3.3 (0.0-6.0) |  | **0.005** | 0.082 | 0.056 |
| **EDEQ-WC** |  |  |  |  |  |  |  |  |  |  |  |  |  |
|  | Mean (SD) | 0.8 (1.2) |  | 4.4 (0.9) |  | 2.5 (2.0) | 3.4 (1.8) |  | 2.5 (2.0) |  |  |  |  |
|  | Median (range) | 0.4 (0.0-3.2) |  | 4.2 (3.4-5.6) |  | 2.2 (0.2-5.6) | 3.5 (0.2-5.6) |  | 2.6 (0.0-5.6) |  | **0.007** | 0.126 | 0.095 |

*Abbreviations:* EDI Eating Disorder Inventory-3, ED-Tot: Total score, ED Risk: Eating Disorder Risk Composite, EDI Psych: Psychological Maladjustment Composite, EDI-DT: Drive for Thinness, EDI-B: Bulimia, EDI-BD: Body dissatisfaction, EDI-LSE: Low Self-esteem, EDI-PA: Personal Alienation, EDI-II: Interpersonal Insecurity, EDI-IA: Interpersonal Alienation, EDI-ID: Interoceptive Deficits, EDI-ED: Emotional Dysregulation, EDI-P: Perfectionism, EDI -A: Asceticism, EDI-MF: Maturity Fears; MADRS-S: Montgomery-Åsberg Depression Rating Scale Self-Assessment; OCI-R: Obsessive-Compulsive-Inventory-Revised; STAI-S/T: State-Trait Anxiety Inventory; EDEQ: Eating Disorders Examination Questionnaire, EDEQ-R: Restraint, EDEQ-EC: Eating Concern, EDEQ-SC: Shape concern, EDEQ-WC: Weight Concern.

**Supplemental table 3**. Individual data for each participant.

| **Group** | **Age** | **BMI** | **Duration (yrs)** | **GLP_1 (pg/mL)** | **Glukagon (pg/mL)** | **GIP (pmol**  **/L)** | **PYY (ng/mL)** |
| --- | --- | --- | --- | --- | --- | --- | --- |
| **Controls** | 29.0 | 24.51 |  | 16.08 | 45.09 | 1.88 | 0.28 |
| **Controls** | 35.4 | 22.14 |  | 15.00 | 19.83 | 57.75 | 0.75 |
| **Controls** | 33.3 | 22.57 |  | 17.53 | 17.50 | 5.06 | 0.40 |
| **Controls** | 27.9 | 26.81 |  | 20.63 | 20.77 | 14.41 | 1.49 |
| **Controls** | 27.5 | 34.00 |  | 15.00 | 26.39 | 3.11 | 0.34 |
| **Controls** | 35.3 | 22.22 |  | 15.00 | 30.35 | 1.88 | 0.24 |
| **Controls** | 34.4 | 35.76 |  | 17.63 | 26.71 | 21.09 | 0.46 |
| **WRAN** | 18.4 | 21.99 | 12 | 19.91 | 18.40 | 57.62 | 0.39 |
| **WRAN** | 29.7 | 19.49 | 9 | 18.36 | 18.63 | 181.99 | 1.09 |
| **WRAN** | 32.9 | 18.91 | 8 | 16.66 | 21.39 | 39.09 | 0.25 |
| **WRAN** | 30.9 | 20.83 | 13 | 15.00 | 25.39 | 1.88 | 0.29 |
| **WRAN** | 26.8 | 23.44 | 9 | 15.82 | 107.34 | 11.86 | 0.28 |
| **non-WRAN** | 34.2 | 15.82 | 22 | 21.48 | 33.97 | 37.01 | 0.66 |
| **non-WRAN** | 30.7 | 17.93 | 16 | 84.94 | 290.00 | 163.47 | 0.72 |
| **non-WRAN** | 24.9 | 15.62 | 13 | 28.84 | 25.78 | 68.21 | 0.56 |
| **non-WRAN** | 21.1 | 17.96 | 7 | 37.44 | 42.40 | 14.14 | 1.33 |
| **non-WRAN** | 28.7 | 17.04 | 14 | 15.00 | 26.61 | 32.20 | 0.80 |

| **Group** | **EDI_Tot** | **EDI_ED Risk** | **EDI-Psych** | **EDI-P** | **Madras** | **OCI_R** | **STAI_S** | **STAI_T** | **EDEQ_Tot** |
| --- | --- | --- | --- | --- | --- | --- | --- | --- | --- |
| **Controls** | 60 | 22 | 38 | 5 | 7 | 10 | 35 | 36 |  |
| **Controls** | 17 | 6 | 11 | 3 | 0 | 5 | 25 | 28 | 0.00 |
| **Controls** | 76 | 14 | 62 | 13 | 15 | 23 | 34 | 45 | 0.63 |
| **Controls** | 51 | 10 | 41 | 9 | 7 | 14 | 29 | 36 | 0.66 |
| **Controls** | 114 | 38 | 76 | 6 |  | 18 | 57 | 55 | 2.51 |
| **Controls** | 45 | 7 | 38 | 5 | 4 | 2 | 37 | 44 | 0.40 |
| **Controls** | 36 | 8 | 28 | 11 | 2 | 2 | 23 | 32 | 0.43 |
| **WRAN** | 145 | 45 | 100 | 12 | 31 | 28 | 49 | 49 | 4.31 |
| **WRAN** | 128 | 40 | 88 | 7 | 10 | 12 |  | 46 | 3.64 |
| **WRAN** | 173 | 65 | 108 | 15 | 33 | 23 | 69 | 71 | 5.00 |
| **WRAN** | 114 | 54 | 60 | 6 | 8 | 22 | 45 | 48 | 4.05 |
| **WRAN** | 150 | 54 | 96 | 8 | 12 | 25 | 47 | 53 | 3.83 |
| **non-WRAN** | 49 | 12 | 37 | 12 | 7 | 8 | 28 | 32 | 0.87 |
| **non-WRAN** | 133 | 29 | 104 | 14 | 25 | 25 | 59 | 58 | 3.70 |
| **non-WRAN** | 102 | 13 | 89 | 21 | 19 | 43 | 67 | 58 | 0.66 |
| **non-WRAN** | 155 | 56 | 99 | 10 | 29 | 31 | 53 | 53 | 4.36 |
| **non-WRAN** | 118 | 41 | 77 | 3 | 21 | 22 | 44 | 48 | 2.09 |

*Abbreviations:* EDI: Eating Disorder Inventory-3; EDI-Tot: Total score; EDI-ED Risk: Eating Disorder Risk Composite; EDI-Psych: Psychological Maladjustment Composite; MADRS-S: Montgomery-Åsberg Depression Rating Scale Self-Assessment; OCI-R: Obsessive-Compulsive-Inventory-Revised; STAI-S/T: State-Trait Anxiety Inventory; EDEQ: Eating Disorders Examination Questionnaire Total score. Patient details separated into controls, those with BMI >18.5 kg/m^2^ (WRAN; weight restored anorexia nervosa) and those with BMI <18.5 kg/m^2^ (non-WRAN; non-weight restored anorexia nervosa).
